# The histaminergic system is involved in psychological stress‐induced hyperthermia in rats

**DOI:** 10.14814/phy2.13204

**Published:** 2017-04-24

**Authors:** Battuvshin Lkhagvasuren, Takakazu Oka

**Affiliations:** ^1^ Department of Psychosomatic MedicineGraduate School of Medical SciencesKyushu UniversityFukuokaJapan; ^2^ The Neuroscience ClusterScience and Technology CenterMongolian National University of Medical SciencesUlaanbaatarMongolia

**Keywords:** Circadian rhythm, histamine, locomotor activity, stress, temperature, thermoregulation

## Abstract

The histaminergic system modulates numerous physiological functions such as wakefulness, circadian rhythm, feeding, and thermoregulation. However, it is not yet known if this system is also involved in psychological stress‐induced hyperthermia (PSH) and, if so, which histamine (H) receptor subtype mediates the effect. Therefore, we investigated the effects of pretreatments with intraperitoneal injections of mepyramine (an H1 receptor inverse agonist), cimetidine (an H2 receptor antagonist), and ciproxifan (an H3 receptor inverse agonist) on cage‐exchange stress‐induced hyperthermia (a model of PSH) by monitoring core body temperature (*T*_c_) during both light (10:00 am–12:00 pm) and dark (10:00 pm–12:00 am) phases in conscious, freely moving rats. We also investigated the effects of these drugs on stress‐induced changes in locomotor activity (*L*_a_) to rule out the possibility that effects on *T*_c_ are achieved secondary to altered *L*_a_. Cage‐exchange stress increased *T*_c_ within 20 min followed by a gradual decrease back to baseline *T*_c_ during both phases. In the light phase, mepyramine and cimetidine markedly attenuated PSH, whereas ciproxifan did not affect it. In contrast, in the dark phase, mepyramine dropped *T*_c_ by 1*°C* without affecting cage‐exchange stress‐induced hyperthermia, whereas cimetidine and ciproxifan did not affect both postinjection *T*_c_ and PSH. Cage‐exchange stress induced an increase in *L*_a_, especially in the light phase, but none of these drugs altered cage‐exchange stress‐induced *L*_a_ in either circadian rhythm phase. These results suggest that the histaminergic system is involved in the physiological mechanisms underlying PSH, particularly through H1 and H2 receptors, without influencing locomotor activity.

## Introduction

Many kinds of psychological stress increase core body temperature (*T*_c_) in mammals such as rats (Briese and de Quijada 1970; Vinkers et al. 2009), mice (Zethof et al. 1995; Oka et al. 2003), rabbits (Yokoi 1966; Snow and Horita 1982), and humans (McNeil et al. 1984; Timmerman et al. 1992; Oka and Oka 2007; Hiramoto et al. 2009; Kaneda et al. 2009). For example, in rats, cage exchange stress, that is, exchanging home cages between two animals (Long et al. 1990a), or social defeat stress, that is, exposure to dominant conspecific animals (Lkhagvasuren et al. 2011), induces a robust increase in *T*_c_ up to 2*°C* within 30 min. This phenomenon is known as psychological stress‐induced hyperthermia (PSH). PSH may have adaptive and beneficial values for animals to survive “fight‐or‐flight” situations because such rapid increases in *T*_c_ in a stressful situation helps to warm up muscular and central nervous systems, leading to increased physical and neurocognitive performance (Wright et al. 2002; Bishop 2003; Kataoka et al. 2014).

Recently, neural mechanisms underlying PSH have been extensively studied. Anatomical studies have demonstrated that, at least in part, the dorsomedial hypothalamus (DMH) – rostral medullary raphe regions (which include the rostral raphe pallidus and the raphe magnus nuclei) – sympathetic nerve axis, is involved in the development of PSH by inducing nonshivering thermogenesis in brown adipose tissue (Cannon and Nedergaard 2004; Lkhagvasuren et al. 2011; Kataoka et al. 2014). Sympathetic nerve‐mediated peripheral vasoconstriction may also contribute to PSH via inhibiting heat loss (Oka et al. 2001). Of note, previous studies have shown that the mechanism responsible for PSH is different from inflammation‐induced fever, which requires proinflammatory mediators (Oka et al. 2003). Pharmacological studies indicated that PSH is not attenuated by systemic administration of cyclooxygenase inhibitors, but is reduced by anxiolytic drugs such as benzodiazepines or serotonin (5‐HT) 1A receptor agonists, *β*3‐adrenoceptor antagonists, or *α*‐adrenoceptor antagonists (Oishi et al. 1992; Nakamori et al. 1993; Groenink et al. 1995; Oka et al. 2001; Lkhagvasuren et al. 2014). So far, however, the role of the histaminergic system in PSH is not known. Furthermore, it has not yet been investigated whether peripheral administration of antihistaminergic drugs affects PSH.

We hypothesized that the histaminergic system is involved in PSH, and that peripheral antihistaminergic drugs would attenuate PSH based on the following findings. First, in our previous study (Lkhagvasuren et al. 2014), we observed that social defeat stress increases *T*_c_ and *Fos* protein expression, a marker of neuronal activation (Sagar et al. 1988), in the tuberomammillary nucleus (TMN), from where histaminergic projections originate, in rats. Furthermore, intraperitoneal (IP) injection of diazepam attenuated both PSH and *Fos* expression in the TMN (Lkhagvasuren et al. 2014). Second, histaminergic neurons in the TMN are involved in arousal (McGinty et al. 2001; Haas et al. 2008; Panula and Nuutinen 2013) and it has been demonstrated that goal‐directed behavioral arousal, such as food anticipation, is accompanied by activation of histaminergic neurons in the TMN and increased *T*_c_ (Valdes et al. 2010).

The histamine (H) receptor has four subtypes, that is, H1, H2, H3, and H4 receptors (Hill et al. 1997). Among them, H1, H2, and H3 receptors are known to be distributed within the brain (Haas et al. 2008; Panula and Nuutinen 2013). However, distribution of H4 receptors in the central nervous system is still controversial (Schneider and Seifert 2016). Therefore, we were determined to elucidate the role of H1, H2, and H3 receptors in PSH by investigating the effects of IP injection of mepyramine (an H1 receptor inverse agonist), cimetidine (an H2 receptor antagonist), and ciproxifan (an H3 receptor inverse agonist) on cage‐exchange stress‐induced hyperthermia in conscious, freely moving rats. In this experiment, we chose cage‐exchange stress because this procedure has been widely accepted as a model of PSH (Long et al. 1990a; Oka et al. 2003), and the magnitude of cage‐exchange stress‐induced PSH is stable and reproducible (Oka et al. 2003). We also assessed the role of H1, H2, and H3 receptors on stress‐induced hyperkinesis because cage‐exchange stress increases locomotor activity (*L*_a_), especially during the light period (Long et al. 1990b). Moreover, some, but not all studies have shown that the histaminergic system modulates locomotor activity (O'Neill and Gertner 1987; Yanai et al. 1998; Toyota et al. 2002). Previous studies have suggested that PSH represents an independent stress response distinct from hyperkinesis (Long et al. 1990b; Houtepen et al. 2011). However, it is not known whether antihistamine drugs can affect *L*_a_ at doses that affect PSH, and vice versa. Therefore, we also investigated the effects of antihistaminergic drugs on *L*_a_ as well as PSH. Since the histaminergic system is involved in the establishment and maintenance of circadian rhythms (Mochizuki et al. 1992), we conducted our experiments during both light and dark circadian phases.

## Materials and Methods

### Animal procedures, diet, and housing

Male Wistar rats weighing 170–190 g (SLC, Kurume, Japan) were used for the following experiments. The rats were individually housed in plastic cages (a cage: 40 cm long × 25 cm wide × 20 cm high) in a room air‐conditioned at 26 ± 1*°C* with a standard 12‐h light–dark cycle (lights on 7:00 am – 7:00 pm) and allowed ad libitum access to food and water. All procedures conformed to the guidelines of animal care by Kyushu University and were approved by the Ethics Committees of Kyushu University.

### Monitoring of *T*_c_ and *L*_a_

We measured *T*_c_ and *L*_a_ of the rats using a telemetry system while the animals were freely moving (Data Sciences International, St. Paul, MN). A battery‐operated telemetric transmitter (TA10TA‐F40) was implanted into the peritoneal cavity of each rat via a midline incision under anesthesia with a mixture (0.1 mL/10 g weight, IP) of medetomidine (0.15 mg/kg), midazolam (2 mg/kg), and butorphanol (2.5 mg/kg). After closure of the cavity with a suture, the rats were housed individually for a week to recover from the surgery under regular health checks. *T*_c_ and *L*_a_ signals were received by an antenna below a rat cage and relayed to a signal processor connected to a server computer. At least 1 day before the experiment, the telemetric transmitters were activated using a magnet to start sampling *T*_c_ (*°C*) and *L*_a_ (counts/min) every 5 min. *L*_a_ counts reflect all movements both in horizontal and vertical directions. Only rats that showed stable circadian rhythm changes in *T*_c_ and *L*_a_ were used for the following experiments. On the experimental day, both *T*_c_ and *L*_a_ were monitored for 24 h.

### Drug injections

On the experimental day, rats received an IP injection of mepyramine (an H1 receptor inverse agonist, 30 mg/kg, 0.15–0.2 mL; Wako, Osaka, Japan [synonym: pyrilamine]), cimetidine (an H2 receptor antagonist, 100 mg/kg, 0.35–0.4 mL; Wako, Osaka, Japan), ciproxifan (an H3 receptor inverse agonist, 10 mg/kg, 0.15–0.2 mL; Sigma, San Francisco), or one of their respective vehicles. To investigate the role of H1 and H3 receptors in the development of PSH, we administered an H1 receptor inverse antagonist and an H3 receptor inverse agonist, respectively. Inverse agonists are molecules that bind to the same receptor as agonists but induce the pharmacological responses opposite to the agonists. Therefore, H1 and H3 inverse agonists have opposite effects to H1 and H3 receptor agonists, respectively. Mepyramine, permeable to the blood‐brain barrier (BBB) via carrier‐mediated transport (Yamazaki et al. 1994), and ciproxifan, also permeable to the BBB (Ligneau et al. 1998), were dissolved in physiological saline, whereas cimetidine, also permeable to the BBB (Whittico et al. 1990), was dissolved first in physiological saline with 0.1N hydrochloric acid, then neutralized with sodium hydroxide (Owen et al. 1980). Since previous studies that investigated the effects of histaminergic drugs on *T*_c_ or *L*_a_ are limited and the results are controversial (Clark and Clark 1980; Clark and Lipton, 1985), we were determined to investigate the effect of a maximal dose of each drug on PSH. Therefore, to begin, we researched the effective dose of each drug (administered intraperitoneally) to investigate their physiological effects in established literature. Then, we investigated the effects of histaminergic drugs at that dose (and higher doses) in freely moving rats in both circadian periods. IP injection of mepyramine at doses greater than 30 mg/kg caused sudden death by abrupt seizures in the dark phase, which was in line with previous studies that demonstrated the proconvulsive effect of H1 antagonists/inverse agonists (Kamei et al. 2000; Haruyama et al. 2008). Therefore, we selected 30 mg/kg for mepyramine (Clark and Clark 1980; Clark and Lipton 1985a; Masuoka et al. 2008). Our pilot study found that the effects of IP injection of cimetidine and ciproxifan at doses widely used in the literature, that is, 30 mg/kg for cimetidine (Clark and Clark 1980) and 3 mg/kg for ciproxifan (Ligneau et al. 1998; Morisset et al. 2000; Fox et al. 2005), and at the maximal doses used in the literature, that is, 100 mg/kg for cimetidine (Hough et al. 1985) and 10 mg/kg for ciproxifan (Esbenshade et al. 2004), on *T*_*c*_ and *L*_*a*_ were similar. Therefore, in this study, we show the results obtained by the higher doses. To minimize the stress of the IP injection procedures, solutions were injected into the lower abdomen, which was exposed to the experimenter by gently bending the lower back backward with the base of the tail lifted up. This procedure was performed within the home cages of the rats while their forelegs were still touching the floor, to avoid moving the head of the rats upside down. Thus, mostly, the PSH following from the IP injection procedure was less than 0.5*°C*, which enabled us to observe the development of subsequent cage‐exchange stress‐induced hyperthermia.

### Cage‐exchange stress

Thirty minutes after the injection, rats were either exposed to cage‐exchange stress (Stress exposure) or left undisturbed in their home cages (Control exposure). The cage‐exchange stress procedure is described in detail elsewhere (Oka et al. 2003). In brief, this was evoked by exchanging the home cages of two rats for 60 min. Thereafter, the rats were returned to their home cages. The experiments were performed between 10:00 am and 12:00 pm in the light phase and between 10:00 pm and 12:00 am in the dark phase, when the circadian rhythm changes in *T*_c_ are minimal. The rats were randomly separated into four groups (*n *=* *4–8 per group) and each group was first IP injected either with a vehicle or drug, then treated either with the control or the stress exposure to produce the following groups: (1) a vehicle injection followed by control exposure (Vehicle/Control group); (2) a vehicle injection followed by stress exposure (Vehicle/Stress group); (3) a drug injection followed by stress exposure (Drug/Stress group); (4) a drug injection followed by control exposure (Drug/Control group).

The average *T*_c_ for a 30‐min period prior to the injection was considered the baseline *T*_c._ The average *T*_c_ for a 30‐min period after the injection was considered the postinjection *T*_c._ In the same way, the average *L*_a_ for a 30‐min period prior to the injection was determined as the baseline *L*_a_. The average *L*_a_ for a 30‐min period after the injection was determined the postinjection *L*_a_.

### Statistical analysis

All data are presented as means ± standard error of the means. The effects of stress/control exposure on *T*_c_ across time were evaluated using two‐way repeated measures ANOVA (groups, time, groups x time) followed by Bonferroni *post hoc* test or unpaired *t*‐test. Differences in baseline *T*_c_ and postinjection *T*_c_ were analyzed using an unpaired *t*‐test or one‐way ANOVA with Bonferroni *post hoc* test, if appropriate. The effects of drugs on postinjection *T*_c_ within a group were analyzed using a paired *t*‐test. The same analyses were completed for *L*_a._ All tests were two‐tailed and results with *P* values of < 0.05 were considered significant (SPSS Statistics, Version 21).

## Results

### The effects of cage‐exchange stress on *T*_c_ and *L*_a_

#### Light phase

**Figure 1:**
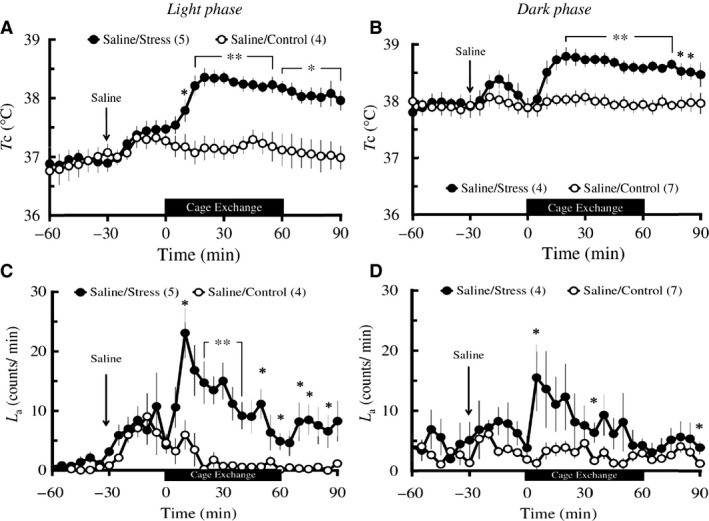
Effects of cage‐exchange stress on *T*_c_ (A and B) and *L*_a_ (C and D) in light and dark phases in rats. Rats (*n *=* *4–7 per group) were administered an IP injection of physiological saline at time ‐30 min (arrows), either exposed to cage‐exchange stress (Stress: filled circles) or left undisturbed (Control: empty circles) at time zero for 60 min (black bar), then returned to their home cages in the light (A and C) and dark (B and D) phases. Each point represents mean ± S.E.M. The differences in both *T*_c_ and *L*_a_ between the two groups were compared using two‐way repeated‐measures ANOVA followed by unpaired *t*‐test (*P <* *0.05, **P <* *0.01). N in the parentheses = number of animals.

Baseline *T*_c_ was not different between Saline/Stress group (*n *=* *5) and Saline/Control group (*n *=* *4). Saline injection did not change the postinjection *T*_c_ in either group and there was no difference in postinjection *T*_c_ between the two groups. However, cage‐exchange stress induced a rapid increase in *T*_c_ in the stressed rats, peaking at 20 min. Two‐way repeated measures ANOVA of the 60 min during the stress/control exposure demonstrated that there was a significant difference in *T*_c_ across time between the two groups (groups: *F*_1_ = 24.68, *P *<* *0.002; time: *F*_2.2_ = 5.71, *P *=* *0.012; groups × time: *F*_2.2_ = 8.07, *P *=* *0.003). Additional *post hoc* analysis using unpaired *t*‐testing of the 90 min including 60 min during the stress/control exposure and subsequent 30 min afterward revealed significant differences from 10 min to 90 min between the two groups (Fig. 1A). Baseline *L*_a_ was not different between the two groups, either. Saline injection induced an increase in the postinjection *L*_a_ in both groups (control: *t*_3_ =  −8.16, *P* = 0.004; stress: *t*_4_ =  −3.04, *P* = 0.016; paired *t*‐test), although there was no difference between the two groups. Cage exchange stress induced a rapid increase in *L*_*a*_ in the stressed rats, peaking at 10 min. Two‐way repeated measures ANOVA revealed that the Saline/Stress group had a significantly greater *L*_a_ (groups: *F*_1_ = 8.61, *P *=* *0.014, time: *F*_4.27_ = 3.01, *P *=* *0.025, groups × time: *F*_4.27_ = 1.97, *P *=* *0.11) during the stress/control exposure across time when compared with the Saline/Control group. The *post hoc* test indicated significant differences at the indicated time points between the two groups (Fig. 1C).

#### Dark phase

Baseline *T*_c_ was not different between Saline/Stress group (*n *=* *4) and Saline/Control group (*n *=* *7). Saline injection did not change the postinjection *T*_c_ in either group and there was no difference in postinjection *T*_c_ between the two groups. Cage exchange stress induced a rapid increase in *T*_c_ in the stressed rats, peaking at 20 min. Two‐way repeated measures ANOVA of the 60 min during the stress/control exposure revealed that the stressed rats had a significantly greater increase in *T*_c_ (groups: *F*_1_ = 14.84, *P *=* *0.004, time: *F*_2.85_ = 4.41, *P *=* *0.013, groups × time: *F*_2.85_ = 9.68, *P *<* *0.001) when compared with the undisturbed rats. Additional *post hoc* analysis using unpaired *t*‐testing of the 90 min including 60 min during the stress/control exposure and subsequent 30 min afterward indicated significantly higher values in *T*_c_ from 20 min to 85 min in the stressed rats versus the undisturbed controls (Fig. 1B). Baseline *L*_a_ was not different between the two groups. Saline injection did not change the postinjection *L*_a_ in either group and there was no difference in postinjection *L*_a_ between the two groups. Two‐way repeated measures ANOVA of the 60 min during the stress/control exposure revealed that there was no difference in *L*_a_ between the two groups, although pairwise comparison at each time‐point with *t*‐testing revealed significant differences at the indicated time points between the two groups (Fig. 1D).

### The effects of antihistaminergic drugs on *T*_c_ and *L*_a_ during postinjection and cage‐exchange stress periods

#### H1 receptor inverse agonist

##### Light phase

**Figure 2:**
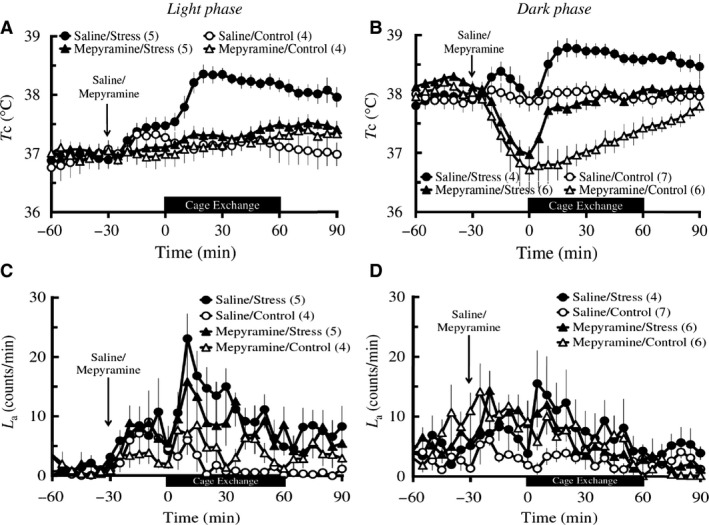
Effects of mepyramine on the cage‐exchange stress‐induced changes in *T*_c_ (A and B) and *L*_a_ (C and D) in light and dark phases. Rats (*n *=* *4–7 per group) were administered with either an IP injection of 30 mg/kg mepyramine (triangles) or physiological saline (circles) at time ‐30 min (arrows), either exposed to cage‐exchange stress (Stress: filled circles) or left undisturbed (Control: empty circles) at time zero for 60 min (black bar), then returned to their home cages in the light (A and C) and dark (B and D) phases. Each point represents mean ± S.E.M. The differences in both *T*_c_ and *L*_a_ among the groups were compared using two‐way repeated measures ANOVA followed by Bonferroni *post hoc* test. N in the parentheses = number of animals.

Baseline *T*_c_ did not differ among the four groups including Saline/Stress (*n *=* *5), Saline/Control (*n *=* *4), Mepyramine/Stress (*n *=* *5), and Mepyramine/Control (*n *=* *4) groups. IP injection of mepyramine did not affect the postinjection *T*_c_ in either the control group or the stress group. Two‐way repeated measures ANOVA of the 60 min during the stress/control exposure followed by Bonferroni *post hoc* testing revealed that mepyramine significantly attenuated the cage‐exchange stress‐induced increase in *T*_c_ (groups: *F*_3_ = 15.4, *P *<* *0.001; time: *F*_2.41_ = 8.17, *P *=* *0.001; groups × time: *F*_7.24_ = 4.12, *P *=* *0.002; Saline/Stress vs. Mepyramine/Stress: *P *=* *0.001; Fig. 2A). Baseline *L*_a_ and postinjection *L*_a_ were not different among the four groups (Fig. 2C). Two‐way repeated measures ANOVA of the 60 min during the stress/control exposure followed by Bonferroni *post hoc* testing revealed that mepyramine did not affect the cage‐exchange stress‐induced increase in *L*_a_ (Mepyramine/Stress vs. Saline/Stress: *P = *1), although there was an overall difference in *L*_a_ among the four groups (groups: *F*_3_ = 4.34, *P *=* *0.017; time: *F*_4.07_ = 4.68, *P *=* *0.002; groups × time: *F*_12.26_ = 1.28, *P *=* *0.246; Fig. 2C).

##### Dark phase

Baseline *T*_c_ did not differ among the four groups including Saline/Stress (*n *=* *4), Saline/Control (*n *=* *7), Mepyramine/Stress (*n *=* *6), and Mepyramine/Control (*n *=* *6) groups. Mepyramine injection caused a marked decrease in postinjection *T*_c_ both in the control (baseline *T*_c_: 38.07 ± 0.06*°C*; postinjection *T*_c_: 37.45 ± 0.23*°C*;* t*_5_ = 3.31, *P *=* *0.021, paired *t‐*test) and the stress group (baseline *T*_c_: 38.21 ± 0.05*°C*; postinjection *T*_c_: 37.64 ± 0.12*°C*;* t*_5_ = 4.61, *P *=* *0.006, paired *t*‐testing). Two‐way repeated measures ANOVA of the 60 min during the stress/control exposure followed by Bonferroni *post hoc* testing revealed that mepyramine significantly decreased *T*_c_ across time (groups: *F*_3_ = 17.19, *P *<* *0.001; time: *F*_2.3_ = 13.14, *P *<* *0.001; groups × time: *F*_6.89_ = 4.82, *P *<* *0.001; Saline/Stress vs. Mepyramine/Stress: *P *=* *0.01; Mepyramine/Control vs. Saline/Control: *P *=* *0.001; Fig. 2B). However, mepyramine did not block the stress‐induced increase in *T*_c_ as Bonferroni *post hoc* testing after two‐way repeated measures ANOVA of the 60 min during the stress/control exposure, showing that there is a difference in *T*_c_ between the stressed and control rats injected with mepyramine (Mepyramine/Stress vs. Mepyramine/Control, *P *=* *0.008). Furthermore, to compare only elevations of *T*_c_ during the stress/control exposure among the groups, Δ*T*_c_ was determined as the difference between the maximum and the nadir of *T*_c_ during the stress/control exposure. There was no difference in Δ*T*_c_ between the two stress groups (Saline/Stress: 0.98 ± 0.14*°C* vs. Mepyramine/Stress: 1.12 ± 0.17*°C*,* P *=* *1). The baseline *L*_a_ was not different among the four groups and IP injection of mepyramine did not affect the postinjection *L*_a_ in either group (Fig. 2D). Two‐way repeated measures ANOVA of the 60 min during the stress/control exposure followed by Bonferroni *post hoc* testing revealed that there was no difference in *L*_a_ across time among the four groups and mepyramine did not affect the cage‐exchange stress‐induced change in *L*_a_ (groups: *F*_3_ = 1.88, *P *=* *0.176; time: *F*_4.27_ = 5.76, *P *<* *0.001; groups × time: *F*_12.82_ = 1.78, *P *=* *0.066; Saline/Stress vs. Mepyramine/Stress: *P *=* *1, Bonferroni post hoc test; Fig. 2D).

#### H2 receptor antagonist

##### Light phase

**Figure 3:**
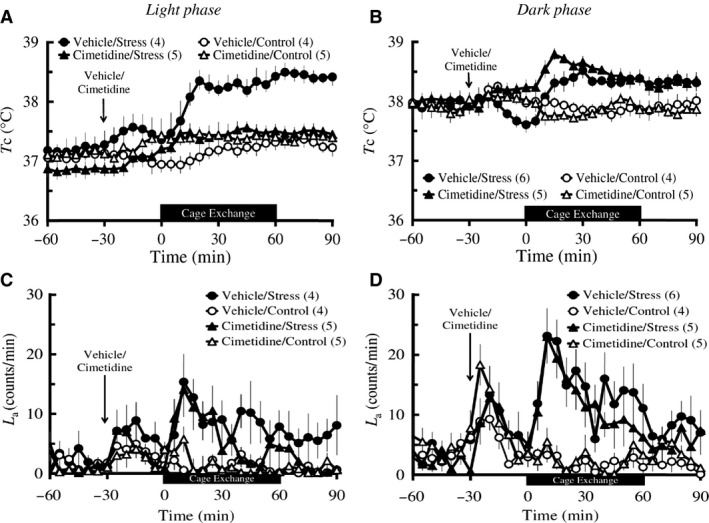
Effects of cimetidine on the cage‐exchange stress‐induced changes in *T*_c_ (A and B) and *L*_a_ (C and D) in light and dark phases. Rats (*n *=* *4–6 per group) were administered with either an IP injection of 100 mg/kg cimetidine (triangles) or its vehicle (circles) at time ‐30 min (arrows), either exposed to cage‐exchange stress (Stress: filled circles) or left undisturbed (Control: empty circles) at time zero for 60 min (black bar), then returned to their home cages in the light (A and C) and dark (B and D) phases. Each point represents mean ± S.E.M. The differences in both *T*_c_ and *L*_a_ among the groups were compared using two‐way repeated measures ANOVA followed by Bonferroni *post hoc* test. N in the parentheses = number of animals.

Baseline *T*_c_ did not differ among the four groups including Vehicle/Stress (*n *=* *4), Vehicle/Control (*n *=* *4), Cimetidine/Stress (*n *=* *5), and Cimetidine/Control (*n *=* *5) groups. IP injection of cimetidine did not affect the postinjection *T*_c_ in either the control group or the stress group. However, two‐way repeated measures ANOVA of the 60 min during the stress/control exposure followed by Bonferroni *post hoc* testing revealed that cimetidine significantly attenuated the cage‐exchange stress‐induced increase in *T*_c_ (groups: *F*_3_ = 8.23, *P *=* *0.002; time: *F*_2.96_ = 11.96, *P *<* *0.001; groups × time: *F*_8.87_ = 3.7, *P *=* *0.002; Vehicle/Stress vs. Cimetidine/Stress: *P *=* *0.028; Fig. 3A). Baseline *L*_a_ and postinjection *L*_a_ were not different among the four groups (Fig. 3C). Two‐way repeated measures ANOVA of the 60 min during the stress/control exposure followed by Bonferroni *post hoc* testing revealed that cimetidine did not affect the cage‐exchange stress‐induced increase in *L*_a_ (Cimetidine/Stress vs. Saline/Stress: *P = *1), although there was an overall difference in *L*_a_ across time among the four groups (groups: *F*_3_ = 3.84, *P *=* *0.034; time: *F*_3.9_ = 5.57, *P *=* *0.001; groups × time: *F*_11.7_ = 2.23, *P *=* *0.023; Fig. 3C).

##### Dark phase

Baseline *T*_c_ did not differ among the four groups including Vehicle/Stress (*n *=* *6), Vehicle/Control (*n *=* *4), Cimetidine/Stress (*n *=* *5), and Cimetidine/Control (*n *=* *5) groups. IP injection of cimetidine did not affect the postinjection *T*_c_ in either the control group or the stress group. Two‐way repeated measures ANOVA of the 60 min during the stress/control exposure followed by Bonferroni *post hoc* testing revealed that cimetidine did not attenuate the cage‐exchange stress‐induced increase in *T*_c_ (groups: *F*_3_ = 17.01, *P *<* *0.001; time: *F*_2.86_ = 4.05, *P *=* *0.013; groups × time: *F*_8.58_ = 4.23, *P *=* *0.001; Vehicle/Stress vs. Cimetidine/Stress: *P *=* *0.102; Fig. 3B). Baseline *L*_a_ and postinjection *L*_a_ were not different among the four groups (Fig. 3D). Two‐way repeated measures ANOVA of the 60 min during the stress/control exposure followed by Bonferroni *post hoc* testing revealed that cimetidine did not affect the cage‐exchange stress‐induced increase in *L*_a_ (Cimetidine/Stress vs. Vehicle/Stress: *P = *1), although there was an overall difference in *L*_a_ among the four groups (groups: *F*_3_ = 11.31, *P *<* *0.001; time: *F*_5.35_ = 5.58, *P *<* *0.001; groups × time: *F*_16.05_ = 2.21, *P *=* *0.009; Fig. 3D).

#### H3 receptor inverse agonist

##### Light phase

**Figure 4:**
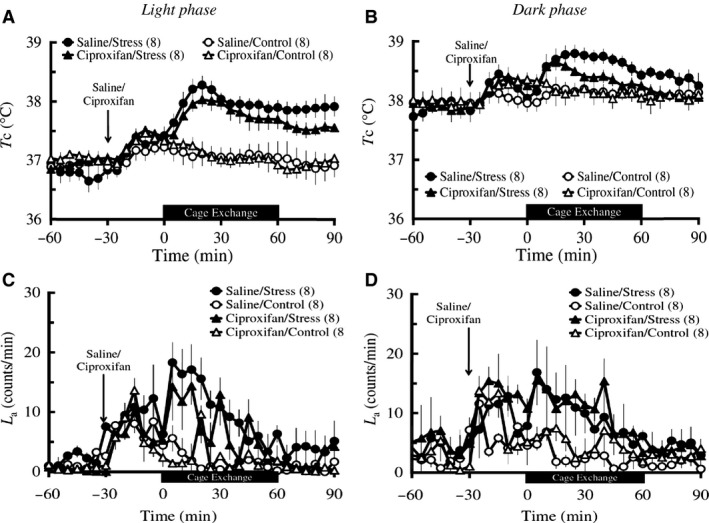
Effects of ciproxifan on the cage‐exchange stress‐induced changes in *T*_c_ (A and B) and *L*_a_ (C and D) in light and dark phases. Rats (*n *=* *8 per group) were administered with either an IP injection of 10 mg/kg ciproxifan (triangles) or its vehicle (circles) at time ‐30 min (arrows), either exposed to cage‐exchange stress (Stress: filled circles) or left undisturbed (Control: empty circles) at time zero for 60 min (black bar), then returned to their home cages in the light (A and C) and dark (B and D) phases. Each point represents mean ± S.E.M. The differences in both *T*_c_ and *L*_a_ among the groups were compared using two‐way repeated measures ANOVA followed by Bonferroni *post hoc* test. N in the parentheses = number of animals.

Baseline *T*_c_ did not differ among the four groups including Saline/Stress (*n *=* *8), Saline/Control (*n *=* *8), Ciproxifan/Stress (*n *=* *8), and Ciproxifan/Control (*n *=* *8) groups. IP injection of ciproxifan did not affect the postinjection *T*_c_ in either the control group or the stress group. Two‐way repeated measures ANOVA of the 60 min during the stress/control exposure followed by Bonferroni *post hoc* testing revealed that ciproxifan did not attenuate the cage‐exchange stress‐induced increase in *T*_c_, although there was an overall difference in *T*_c_ among the groups (groups: *F*_3_ = 18.75, *P *<* *0.001; time: *F*_1.3_ = 9.05, *P *=* *0.022; groups × time: *F*_4.1_ = 5.9, *P *=* *0.032; Saline/Stress vs. Ciproxifan/Stress: *P *=* *0.327; Fig. 4A). Baseline *L*_a_ and postinjection *L*_a_ were not different among the four groups (Fig. 4C). Two‐way repeated measures ANOVA of the 60 min during the stress/control exposure followed by Bonferroni *post hoc* testing revealed that ciproxifan did not affect the cage‐exchange stress‐induced increase in *L*_a_ (Ciproxifan/Stress vs. Saline/Stress: *P = *0.505), although there was an overall difference in *L*_a_ across time among the four groups (groups: *F*_3_ = 6.78, *P *=* *0.006; time: *F*_2.8_ = 3.45, *P *=* *0.056; groups × time: *F*_8.4_ = 1.15, *P *=* *0.405; Fig. 4C).

##### Dark phase

Baseline *T*_c_ did not differ among the four groups including Saline/Stress (*n *=* *8), Saline/Control (*n *=* *8), Ciproxifan/Stress (*n *=* *8), and Ciproxifan/Control (*n *=* *8) groups. IP injection of ciproxifan did not affect the postinjection *T*_c_ in either the control group or the stress group. Two‐way repeated measures ANOVA of the 60 min during the stress/control exposure followed by Bonferroni *post hoc* testing revealed that ciproxifan did not attenuate the cage‐exchange stress‐induced increase in *T*_c_, although there was an overall difference in *T*_c_ among the groups (groups: *F*_3_ = 4.61, *P *=* *0.087; time: *F*_2.1_ = 3.77, *P *=* *0.066; groups × time: *F*_6.4_ = 2.73, *P *=* *0.088; Saline/Stress vs. Ciproxifan/Stress: *P *=* *1; Fig. 4B). Baseline *L*_a_ was not different among the four groups (Fig. 4D). IP injection of ciproxifan did not affect the postinjection *L*_a_ in either the control group or the stress group. Two‐way repeated measures ANOVA of the 60 min during the stress/control exposure followed by Bonferroni *post hoc* testing revealed that ciproxifan did not attenuate the cage‐exchange stress‐induced increase in *L*_a_, although there was an overall difference in *L*_a_ among the groups (groups: *F*_3_ = 5.11, *P *=* *0.045; time: *F*_3.2_ = 8.78, *P *=* *0.002; groups × time: *F*_9.8_ = 1.41, *P *=* *0.268; Saline/Stress vs. Ciproxifan/Stress: *P *=* *1; Fig. 4D).

## Discussion

This study demonstrates that the histaminergic system is involved in the development of PSH, especially through H1 and H2 receptor systems. It also suggests that the roles of these receptors in PSH are different depending on circadian phase.

In the light phase, mepyramine, an H1 receptor inverse agonist, and cimetidine, an H2 receptor antagonist, attenuated cage‐exchange stress‐induced hyperthermia without affecting basal *T*_c_. In contrast, ciproxifan, an H3 receptor inverse agonist, has no effect on the cage‐exchange stress‐induced hyperthermia or basal *T*_c_. It was demonstrated that the DMH‐rostral medullary raphe regions‐sympathetic nerve axis plays an important role in the development of PSH (Lkhagvasuren et al. 2011; Kataoka et al. 2014). In contrast, the involvement of the preoptic area of the hypothalamus (POA), a thermoregulatory center, in the PSH has not been elucidated yet, whereas the POA plays a crucial role in the development of fever when animals suffer from infectious diseases by disinhibiting the DMH‐rostral medullary raphe regions‐sympathetic nerve axis (Nakamura 2011; Saper et al. 2012; Morrison et al. 2014). So far, it is not known if histamine directly affects neuronal activities in the DMH or the rostral medullary raphe regions to induce PSH. In contrast, several studies have suggested that histamine acts on the neurons in the POA to induce hyperthermia. For example, in mice, intra‐POA injection of histamine, 2‐pyridylethylamine (an H1 receptor agonist), and R‐*α*‐methylhistamine (an H3 receptor agonist) increased *T*_c_ without affecting locomotor activity (Lundius et al. 2010). Another study demonstrated that, in mice, intra‐POA injection of 2‐pyridylethylamine and dimaprit (an H2 receptor agonist) increased *T*_c_ without affecting locomotor activity, whereas R‐*α*‐methylhistamine did not affect *T*_c_ (Tabarean et al. 2012). In rats, intra‐POA injection of cimetidine attenuated hyperthermia induced by intracerebroventricular (ICV) injection of histamine (Colboc et al. 1982). These findings suggest that H1 and H2 receptors in the POA mediate the hyperthermic effects of histamine, despite the fact that the role of H3 receptors appears inconclusive. Considering that many kinds of stress activate histaminergic neurons in the TMN (Miklos and Kovacs 2003), it is possible that the histaminergic neurons projecting from the TMN to the POA contribute to the development of PSH via H1 and H2 receptors.

Many kinds of psychological stress, including cage‐exchange stress, increase arousal level in animals. Recently, motivated arousal, which was evoked by enticing hungry animals with food, was demonstrated to increase *T*_c_ by activating the infralimbic cortex (IL) and the TMN (Valdes et al. 2010; Riveros et al. 2014, 2015). Furthermore, IL activation‐induced hyperthermia was attenuated by ICV injection of mepyramine (Riveros et al. 2014). These findings suggest that H1 receptors play an important role in increased arousal‐associated hyperthermia as well as PSH.

In the dark phase, however, the effects of mepyramine and cimetidine on basal *T*_*c*_ or PSH were not the same as effects in the light phase. First, mepyramine decreased basal *T*_*c*_ that prevented the maximum increase in *T*_*c*_ to remain within the normothermic range, although it did not block the cage exchange‐induced increase in *T*_*c*_ in the dark phase (Fig. 2B). However, it inhibited PSH without affecting basal *T*_*c*_ in the light phase. Second, cimetidine did not affect the cage exchange‐induced hyperthermia in the dark phase, whereas it attenuated PSH in the light phase. So far, the precise mechanisms for these differences are not known. However, these phenomena, at least in the case of H1 receptor‐mediated effects, may be associated with characteristics of the TMN neurons, whose firing rate is increased during the dark phase and decreased or absent during the light phase (Mochizuki et al. 1992; Takahashi et al. 2006). In accordance with this, histamine levels in the anterior hypothalamus and locomotor activity are higher in the dark phase than the light phase (Mochizuki et al. 1992; Takahashi et al. 2006). Mepyramine was demonstrated to decrease wakefulness and increase deep slow wave sleep (Lin et al. 1988), suggesting the involvement of H1 receptors in wakefulness. Furthermore, given the involvement of H1 receptors in arousal‐associated hyperthermia into account, it is reasonable to think that mepyramine reduced *T*_*c*_ in the dark phase down to that of the basal level observed in the light phase (around 37.0*°C*).

We also investigated the effects of antihistaminergic drugs on stress‐induced hyperkinesis. Baseline activity was higher in the dark phase versus the light phase. None of the drugs we administered affects *L*_*a*_ in either circadian phase, at least with the doses we tested. These findings are in agreement with the majority of previous studies demonstrating that antihistaminergic drugs do not affect locomotor activity in either phase (Sakai et al. 1991; Imaizumi et al. 1996; Perez‐Garcia et al. 1999), despite several conflicting results (O'Neill and Gertner 1987; Riveros et al. 2014). Therefore, the inhibitory effects of mepyramine and cimetidine on PSH are not likely due to decreased locomotor activity.

There are several limitations of this study. First, we investigated the role of H receptors in PSH via IP injection of H1, H2, and H3 receptor antagonists/inverse agonists. In this study, we administered mepyramine, an H1 receptor inverse agonist, to assess the role of H1 receptors. It is because highly selective H1 antagonists are not available and its apparent constitutive activity of H1 receptors is low (Seifert et al. 2013), so the inverse agonist, mepyramine likely served as an H1 antagonist exerting a limited inverse‐agonistic effect.

One of the reasons we injected drugs systemically was to explore the possibility of these drugs being a translational treatment of psychogenic fever in humans (Oka et al. 2001; Oka 2015). However, there remains a possibility that systemic injection of H receptor antagonists/inverse agonists affects the thermoregulatory responses according to their peripheral actions (such as vasodilation), especially through H1 and H2 receptors (Owen et al. 1980). In the current set of experiments, this is unlikely because, if this is the case, IP injection of mepyramine or cimetidine should decrease the postinjection *T*_*c*_ in the light phase. To negate the possibility of peripheral actions fully, these findings should be reconfirmed by ICV injection of these drugs. Secondly, although this study strongly suggests that the histaminergic system is involved in the development of PSH, its underlying mechanisms are not fully understood, especially as to how the histaminergic system affects the DMH‐rostral medullary raphe regions‐sympathetic axis.

In conclusion, this study demonstrated that the histaminergic system is involved in PSH, especially via H1 and H2 receptors. However, the role of these receptors in the development of PSH may be different depending on the circadian phase.

### Clinical implication and significance

This study provides a substantial contribution to the understanding of the thermoregulatory mechanisms underlying autonomic and behavioral responses to psychological stress. Furthermore, the present findings suggest new therapeutic agents, antihistaminergic drugs, to alleviate stress‐related disorders such as psychogenic fever (or functional hyperthermia), which is characterized by an antipyretic drug‐resistant intense or long‐lasting hyperthermia in stressful situations that is accompanied by an impaired quality of life (Timmerman et al. 1992; Oka and Oka 2007; Lkhagvasuren et al. 2013; Oka 2015).

## Conflict of Interest

None declared.
